# Supplementary material for: Evidence on access to healthcare information by women of reproductive age in low- and middle-income countries: Scoping review
Source: PLoS One. 2021 Jun 4;16(6):e0251633. doi: 10.1371/journal.pone.0251633 (PMC8177524; doi:10.1371/journal.pone.0251633)
Supplement: S1 Table — (DOCX) [file pone.0251633.s001.docx]

|  | Criteria | Determinants |
| --- | --- | --- |
| P | Population | - Women of reproductive age in LMICs |
| C | Concept | - Any interventions that enable women of reproductive age to access healthcare information carried out during 2004 to the present |
| C | Context | - Research articles are limited to LMICs - All languages will be included - Studies conducted as from 2004 to the present will be included |

**S1 Table: PCC Framework**
