# Supplementary material for: Evidence on access to healthcare information by women of reproductive age in low- and middle-income countries: Scoping review
Source: PLoS One. 2021 Jun 4;16(6):e0251633. doi: 10.1371/journal.pone.0251633 (PMC8177524; doi:10.1371/journal.pone.0251633)
Supplement: S1 Fig — (DOCX) [file pone.0251633.s002.docx]

**S1 Fig: Evidence based-framework fro access and utilization of maternal and child health information by adolescents girls during pregnancy**
