# Supplementary material for: Evidence on access to healthcare information by women of reproductive age in low- and middle-income countries: Scoping review
Source: PLoS One. 2021 Jun 4;16(6):e0251633. doi: 10.1371/journal.pone.0251633 (PMC8177524; doi:10.1371/journal.pone.0251633)
Supplement: S2 Table — (DOCX) [file pone.0251633.s003.docx]

**S2 Table: Results for individual sources of evidence**

| **Author and date** | **Study title** | **Study design** | **Study setting (country)** | **Geographic setting (rural/urban)** | **Study population** | **Age** | **% of females** |
| --- | --- | --- | --- | --- | --- | --- | --- |
| Han, 2018 | Progress towards universal health coverage in Myanmar | Quantitative stratified multistage design | Myanmar | Both | Demographic health survey (DHS) data and Integrated household living condition assessment. | Not indicated | Not indicated |
| LeFevre, 2018 | Forecasting the value for money of mobile maternal health | Qualitative-retrospective case control study | Gauteng, South Africa | Both | Pregnant women | 14-49 | 100 |
| Mayora, 2014 | Incremental cost of increasing access to maternal health | Quasi-experimental voucher study | Eastern Uganda | Rural | Two districts (three health sub-districts each) | 14-49 | 100 |
| Parajuli, 2017 | Exploring the role of telemedicine in improving access to healthcare services by women and girls in rural Nepal | Mixed method | Nepal | Rural | Girls and women | 17-37 | 100 |
