# Supplementary material for: Evidence on access to healthcare information by women of reproductive age in low- and middle-income countries: Scoping review
Source: PLoS One. 2021 Jun 4;16(6):e0251633. doi: 10.1371/journal.pone.0251633 (PMC8177524; doi:10.1371/journal.pone.0251633)
Supplement: S3 Table — (DOCX) [file pone.0251633.s004.docx]

**S3 Table: Data base search**

| **Date** | **Database** | **Key search words** | **Number of articles found** | **Number of articles found eligible** |
| --- | --- | --- | --- | --- |
| 18/8/2018 | PubMed | Access and healthcare services | 84 889 | 205 |
| 18/8/2018 | EBSCOhost | Access and healthcare services | 31 825 | 90 |
| 18/8/2018 | EBSCOhost | Access and healthcare services | 30 297 | 213 |
| 18/8/2018 | Google Scholar | Access and healthcare services | 153 000 | 68 |
| 18/8/2018 | Emerald | Access and healthcare services | 74 501 | 32 |
| 19/08/2018 | EBSCOhost | Access and healthcare services and Lower and Middle Income Countries | 72 | 19 |
| 19/08/2018 | Emerald | Access and healthcare services and Lower and Middle Income Countries | 535 | 11 |
| 19/08/2018 | Google Scholar | Access and healthcare services Lower and Middle Income Countries | 2100 | 81 |
| 19/08/2018 | PubMed | Access and healthcare services and Lower and Middle Income Countries | 238 | 31 |
|  |  | Total | **377 457** | 750 |
|  |  | Articles deleted | **-** | 76 |
|  |  |  | **=** | 674 |
|  |  | Duplicates removed | **-** | 65 |
|  |  | Total eligible | **=** | 609 |
