# Supplementary material for: Evidence on access to healthcare information by women of reproductive age in low- and middle-income countries: Scoping review
Source: PLoS One. 2021 Jun 4;16(6):e0251633. doi: 10.1371/journal.pone.0251633 (PMC8177524; doi:10.1371/journal.pone.0251633)
Supplement: Attachment — Submitted filename: Comments to the Authors.docx [file pone.0251633.s005.docx]

**Guest Editor’s Recommendations**

Line 59--- MCHI—please write this in full followed by this acronym in brackets

Line 71-74---the aim of the study as stated may be challenging to the reader regarding its understanding; the following simplified version may be used for better clarity

**The main aim of this study was to evaluate the accessibility to healthcare information for women of reproductive age in low and middle-income countries (LMIC). This was done by undertaking a literature search for available interventions which enable reproductive age women in LMIC access healthcare information.**

Lines 74-78---difficult to understand and actually not needed here so recommend deleting

86-91---the interventions of interest in this study were health promotion (NOT healthcare promotion)…………

Materials and Methods…should all be written in the past tense; currently, it is a mixture of present, past, ad future tenses.

For greater clarity, the part talking about the scoping review protocol can be re-phrased as follows:

This scoping review was guided by Trico et al (year) and followed the the Preferred Reporting Items for Systematic Reviews and Meta-Analyses Extensions for Scoping Reviews (PRISMA-ScR) Protocol. The following are components of a PRISMA review protocol……..

The main subheadings under the Materials and Methods should be differentiated from the sub-divisions under them. This can be done by the use of different font sizes or other formatting features including italics, bolding etc
