# Supplementary material for: Evidence on access to healthcare information by women of reproductive age in low- and middle-income countries: Scoping review
Source: PLoS One. 2021 Jun 4;16(6):e0251633. doi: 10.1371/journal.pone.0251633 (PMC8177524; doi:10.1371/journal.pone.0251633)
Supplement: Attachment — Submitted filename: Responses to Reviewers.docx 18 April 2021.docx [file pone.0251633.s006.docx]

**Reviewers’ Responses**

**Reviewer’s comment:** Line 59--- MCHI—please write this in full followed by this acronym in brackets

**Response:** The acronym MCHI has been written in full. Kindly see page 3, Line 55

**Reviewer’s comment:** Line 71-74---the aim of the study as stated may be challenging to the reader regarding its understanding; the following simplified version may be used for better clarity

**The main aim of this study was to evaluate the accessibility to healthcare information for women of reproductive age in low and middle-income countries (LMIC). This was done by undertaking a literature search for available interventions which enable reproductive age women in LMIC access healthcare information.**

**Response:** The aim of the study has been rephrased as per the recommendation. Kindly see page 3, Line 68 – 73.

**Reviewer’s comment:** Lines 74-78---difficult to understand and actually not needed here so recommend deleting

**Response:** Line 74 – 78 has been deleted as per the recommendation. Please see page 4.

**Reviewer’s comment:** 86-91---the interventions of interest in this study were health promotion (NOT healthcare promotion)…………

**Response:** Health promotion has been inserted accordingly as per the recommendation. Kindly see page 4. Line 82 - 83.

**Reviewer’s comment:** Materials and Methods…should all be written in the past tense; currently, it is a mixture of present, past, ad future tenses.

**Response:** Materials and Methods has been edited to past tense accordingly as per the recommendations. Kindly see section materials and methods.

**Reviewer’s comment:** For greater clarity, the part talking about the scoping review protocol can be re-phrased as follows:

This scoping review was guided by Trico et al (year) and followed the Preferred Reporting Items for Systematic Reviews and Meta-Analyses Extensions for Scoping Reviews (PRISMA-ScR) Protocol. The following are components of a PRISMA review protocol……..

**Response:** The section on scoping review protocol has been revised as per the recommendations. Kindly see page 4, Line 87 – 89.

**Reviewer’s comment:** The main subheadings under the Materials and Methods should be differentiated from the sub-divisions under them. This can be done by the use of different font sizes or other formatting features including italics, bolding etc

**Response:** The subheadings under Materials and Methods has been differentiated from the subdivisions by giving different fonts. Materials and methods is font 18, the subheadings are Font 16 and sub-divisions are font 14.
