# Supplementary material for: Evidence on access to healthcare information by women of reproductive age in low- and middle-income countries: Scoping review
Source: PLoS One. 2021 Jun 4;16(6):e0251633. doi: 10.1371/journal.pone.0251633 (PMC8177524; doi:10.1371/journal.pone.0251633)
Supplement: Attachment — Submitted filename: Responses to Reviewers.docx 27 April 2021.docx [file pone.0251633.s007.docx]

**Reviewers’ Responses**

**Reviewer’s comment:** Fig 1 is a Schematic diagram of the selection process for the studies used for the scoping review and should be titled as such because the current title of Fig 1 is not appropriate.

**Response:** Thank you very much for the comment. The title for Figure 1 has been revised and inserted as recommended and it is now reading: **“Schematic diagram of the selection process for the studies used for the scoping review”.**  Kindly see figure 1, page 11.

**Reviewer’s comment:** Under the Discussion - Summary of Evidence---lines 6-8 the sentence is better stated as follows:

Women with high education have greater access to healthcare information than those with lower education.

**Response:** Thank you very much for the comment, the sentence has been revised to read as “**Women with high education have greater access to healthcare information than those with lower education**”. Kindly see page 17, Line 6-8

**Reviewer’s comment:** Under limitations -- line 9 on page 20
------randomized control trials to assess strategies aimed at enabling these women to access------ (note that the correct word to use before strategies is "assess" and not "access")

**Response:** Thank you very much for the comment, the word “access” has been revised to read as “assess” as recommended. Please see page 20, Line 9.
